# Advancing In Vitro Microfluidic Models for Pressure-Induced Retinal Ganglion Cell Degeneration: Current Insights and Future Directions from a Biomechanical Perspective

**DOI:** 10.3390/mi16121368

**Published:** 2025-11-30

**Authors:** Tianyi Gao, Junhao Hao, Heather Mak, Zhiting Peng, Jing Wu, Qinyu Li, Yau Kei Chan

**Affiliations:** 1 Tanwei College, Tsinghua University, Beijing 100084, China; 2 Department of Ophthalmology, LKS Faculty of Medicine, The University of Hong Kong, Hong Kong 999077, China; u3597474@connect.hku.hk (J.H.); u3007594@connect.hku.hk (Z.P.); 3 Department of Mechanical Engineering, The University of Hong Kong, Hong Kong 999077, China

**Keywords:** glaucoma, intraocular pressure, retinal ganglion cell degeneration, microfluidics, biomechanics

## Abstract

Glaucoma is the leading cause of irreversible blindness, primarily characterized by retinal ganglion cell (RGC) loss and optic nerve damage due to abnormal alterations in intraocular pressure (IOP). While in vivo models provide valuable insights into its pathophysiology, they face limitations in controlling biomechanical parameters and long-term IOP monitoring. In vitro models offer greater experimental control but often lack the complexity of the ocular microenvironment, limiting their physiological relevance. To better understand RGC degeneration from a biomechanical perspective, advancements are needed to improve these models, including precise pressure manipulation and more realistic cell culture conditions. This review summarizes current in vitro approaches for studying pressure-induced RGC degeneration and explores the potential of microfluidic technologies to enhance model fidelity. Incorporating microfluidic technologies holds promise for creating more physiologically relevant models, potentially advancing our understanding of IOP-related RGC degeneration from biomechanical perspectives.

## 1. Introduction

Glaucoma is the leading cause of irreversible blindness worldwide, and the total number of glaucoma patients is estimated to reach 110 million by 2040 [1]. This disease is defined as a degenerative optic neuropathy with a hallmark of progressive loss of retinal ganglion cells (RGCs). Glaucoma has many sub-types with many possible causes [2], of which elevated intraocular pressure (IOP) is the most well-known and established risk factor across clinical and experimental studies of glaucoma [3,4,5]. IOP is regulated by the balance between production and drainage of aqueous humor in the eye [6], which is continuously produced by the ciliary body [7]. The drainage system of the eye, also known as the aqueous outflow system, is crucial in maintaining a normal IOP, as the amount of fluid drained from the eye has to balance that of what the ciliary body produces. The impairment of the outflow system will result in building up of aqueous fluid, hence raised IOP, and eventually glaucoma.

In vivo animal models are crucial in studies on the pathogenesis and drug developments for glaucoma [8,9,10,11,12,13,14,15]. However, despite previous and ongoing in vivo studies on both cellular and biomolecular aspects of glaucoma [2,3,8,16,17,18], the inherent limitations of current in vivo models hinder their use to study the effect of IOP-related RGC degeneration. Most glaucoma patients have mild to moderate IOP elevation, with chronic and progressive loss of RGCs [19]. However, many of the existing animal glaucoma models induce high IOP spike leading to ischemic damage of the optic nerve instead [20]. Moreover, the levels of IOP elevation are difficult to consistently adjust in animal models [2], since IOP fluctuates throughout the day owing to both the circadian rhythm and cardiac rhythm [21,22,23]. Real-time IOP measurement is even more technically challenging due to the active motion of animals [2]. Studying the effects of elevated pressure levels and pressure fluctuation amplitudes on RGC degeneration requires a system capable of consistently and reliably controlling these parameters.

To address the abovementioned research gap, some studies have focused on developing in vitro models to serve as complementary tools to in vivo models. The most widely reported among these include static and dynamic pressure models. Static pressure models are more established compared to their dynamic counterparts. They employ pressurized chambers [24,25,26,27,28,29,30,31,32,33], liquid-column systems [34,35,36], centrifugal force loading [37], or pressurized flasks [38] to apply consistent pressure to RGCs. However, the pressure levels tested vary significantly across studies, making comparative analysis challenging. Dynamic pressure models hold greater promise as they can simulate the fluctuating IOP observed in glaucoma patients more accurately. Nevertheless, further research is necessary to validate their effectiveness and their ability to replicate an ideal pathological environment [39,40]. Besides the capacity to precisely generate variable pressure levels, there is a need for more diverse models, ranging from single RGCs to retinal organoids, to comprehensively investigate all factors contributing to pressure-induced degeneration of RGCs. While RGC-based models primarily focus on the direct mechanical compression resulting from elevated IOP, models incorporating other retinal cell types can help elucidate the role of biochemical stimuli triggered by ocular tissue deformation under high-pressure conditions.

With advances in microfabrication technologies, current in vitro microfluidic models show significant potential in meeting the aforementioned expectations for in vitro systems. They not only facilitate the study of IOP-related RGC degeneration in glaucoma for future drug development and disease modeling, but also provide researchers with an effective platform to investigate other potential mechanisms of IOP-induced pathology. In this review, we focus on reviewing in vitro models in the literature that aim to address research questions related to pressure-induced degeneration of RGCs and the possible result of sight-threatening glaucoma. We then suggest further applications of microfluidic technologies and improvements in the design of the microfluidic models for identifying pathogenesis of glaucoma from biomechanical perspectives.

## 2. Pressure Modes in Current In Vitro Studies on Pressure-Induced Degeneration of RGCs: Static or Dynamic?

In vitro models with pressure control can address some of the aforementioned limitations of in vivo models with respect to studies about pressure-induced degeneration of RGCs. In vitro hydrostatic pressure models offer higher flexibility, more precise control of cell culture environments, and higher reproducibility in comparison to in vivo models [24,25,26,41]. The use of in vitro models opens up the possibility of studying cellular and molecular mechanisms related to the degeneration of RGCs with lower complexity, and provides insight into understanding the development of glaucoma. These models can be used to study the cellular response under static or dynamic pressure profiles [27,28,29,30,31,32,33,34,39,40].

### 2.1. Static Pressure Models

**Figure 1:**
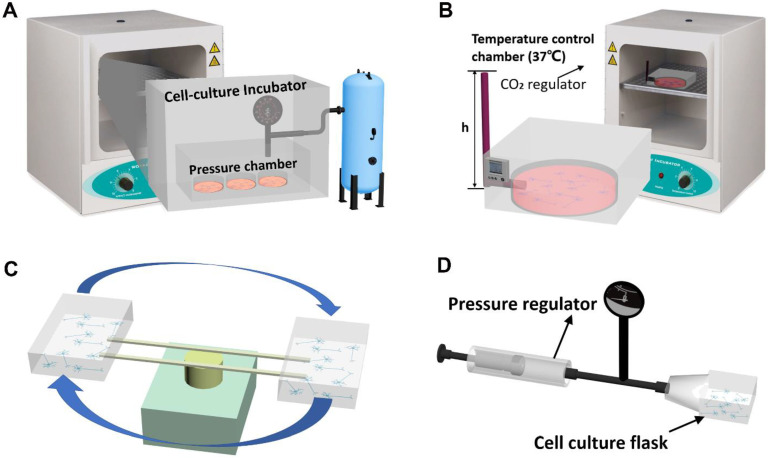
Schematics of current in vitro static pressure platforms. (**A**) Pressurized chamber model [27,28,30,31,32,33,34,39,40,42], (**B**) liquid-column pressure model [29,43,44], (**C**) centrifugal force loading model [37], and (**D**) pressurized flask model [38].

Static pressure models, which consist of a stable and static pressure source that can elevate the pressure within a sealed compartment, are more commonly used than dynamic pressure models for studying the pressure-induced degeneration of RGCs. The static pressure models can be classified into pressurized chamber models [27,28,30,31,32,33,34,39,40,42], liquid-column models [29,43,44], centrifugal force loading models [37], and pressurized flask models [38] (Figure 1A–D). In both pressurized chamber models and cell culture flask models, the pressure is elevated by compressed air and controlled by pressure valves [27,28,30,31,32,33,34,39,40,42]. In liquid-column pressure models, the pressure is applied and controlled by the liquid height of the connecting reservoir [29,35,36,43,44,45,46]. In centrifugal force loading models, the pressure is elevated and controlled by centrifugal forces [37]. These systems are maintained in standard cell culture conditions at a constant temperature (37 °C) and carbon dioxide level (5% CO_2_) in humidified culture chambers. However, the amplitude of the elevated pressure varies significantly among all studies, ranging from 7.4 to 100 mmHg. Hence, the non-standardized pressure levels being tested in the literature render the results between the published studies non-comparable.

### 2.2. Dynamic Pressure Models

Dynamic pressure models consist of a dynamic pressure source that can alter the pressure within a sealed compartment, and therefore recreate an in vitro system with dynamic pressure changes. Although static pressure models can aid in studies on RGC degeneration, dynamic pressure models are more physiologically representative as they can mimic IOP, which is fluctuating in nature. IOP is dynamic and pulsatile, due to both the circadian cycle [22,23,47] and pulsatile ocular blood flow (POBF) [48,49]. Both the amplitude and frequency of fluctuating IOP are associated with the pathogenesis of glaucoma [50,51,52,53,54,55], but its exact role remains elusive. Currently, only limited in vitro studies on RGCs have been conducted using dynamic pressure profiles [39,40], likely because of the engineering difficulties encountered during establishment of platforms with precise control over dynamic parameters. However, these studies provided important insights into dynamic pressure in the degeneration of RGCs. For example, in contrast to the static pressure study, a transient pulse of 50 mmHg for 1 min can already have detrimental effects on RGCs, suggesting that RGCs are also sensitive to pressure rhythms in addition to absolute IOP levels [39,40]. However, the pressure rhythms used in these studies were not physiologically representative, as both the amplitudes and frequencies were not comparable with the IOP profile. The amplitudes of pressure fluctuation in these studies (50 and 90 mmHg) were much exaggerated [39,40] compared to those in typical human subjects, which range between 0.9 and 7.2 mmHg [56]. The frequency of the applied pressure rhythms should also mimic that of POBF between 1 and 2 Hz (referring to 60 to 120 beats per minute). Therefore, to study the pressure-induced degeneration of RGCs in future in vitro studies, a dynamic pressure system that closely imitates the abovementioned fluctuations with appropriate physiologically relevant amplitude and frequency should be used.

## 3. The Direct and Indirect Pressure Effect on Pressure-Induced Degeneration of RGCs

The pressure effect on RGCs was studied either by (1) direct application of pressure onto RGCs, or (2) indirect application of pressure via other retinal cells neighboring the RGCs. Their advantages and limitations in studying the pressure-induced degeneration of RGCs are also discussed below.

### 3.1. Direct Pressure Effect on RGCs

The in vitro pressurized systems for studying the pressure-induced degeneration of RGCs by direct application of pressure to RGCs are summarized in Table 1. Currently, representative cell lines of RGCs are lacking [57]. According to studies using the cell line RGC-5, elevated hydrostatic pressure could induce apoptosis, oxidative stress, mitochondrial changes, and cellular ATP reduction on this cell line [27,28]. However, RGC-5 has been abandoned in vision science research due to reports of their lack of RGC-specific genes and proteins [58] and contamination of cultures with another photoreceptor cell line, cell line 661 W [59]. Therefore, the results obtained and concluded from the two studies have to be scrutinized and re-evaluated carefully. In vitro studies on degeneration of RGCs now focus on primary RGCs, which provides more realistic and physiological cellular responses [60,61,62,63]. Among these studies, elevated pressures can induce reduction in axonal length, segmentation along neurites [35,37], and apoptosis of RGCs, possibly by activation of Capsaicin-sensitive vanilloid subunite1 (TRPV1) [32,33]. However, culturing primary RGCs in vitro is technically challenging, due to possible contamination by other cell types resulting from suboptimal cell isolation methods [64]. Recently, the use of the two-step immuno-magneto-panning (TIMP) method has been demonstrated to improve the isolation purity of RGCs [65], which may facilitate studies on the pathophysiology of glaucoma based on in vitro RGCs models.

### 3.2. Indirect Pressure Effect on RGCs

Although RGCs are known to be sensitive to elevated IOP, other cell types in the visual system also respond to elevated IOP which eventually affect RGCs. For example, the response of RGCs to elevated IOP in glaucoma likely involves signals from astrocytes and microglia [67,68]. The in vitro responses of other retinal cells to elevated pressure, which are in close proximity to RGCs, are summarized in Table 2. Elevated pressure can induce significant apoptosis in neural cell line B35, a cell line derived from the central nervous system of rats [34]. An increase in pressure can trigger calcium release from rat optic nerve astrocytes, which subsequently leads to ERK1/2 phosphorylation, which may explain part of the glaucomatous optic nerve damage [43]. Upon elevated pressure, Müller cells in the retina upregulate glutamine synthetase, an enzyme that converts glutamate and ammonia to glutamine, which can lead to neuronal damage at high concentrations [38]. In a tri-culture model comprising primary RGCs, astrocytes, and microglia from rat, Interleukin-6 (IL-6) secreted by both astrocytes and microglia significantly improved RGC survival under elevated pressure [32]. Another study using retinal organotypic cultures showed that the blockade of the adenosine A2A receptor (A2AR) reduces microglia-mediated retinal neuroinflammation and protects RGCs under elevated pressure [30]. All of the above studies demonstrate that elevated pressure not only induces degeneration of RGCs directly, but also triggers various biological responses of the neighboring cells of the retina, which ultimately affects the survival of RGCs. To better investigate the coordinated responses of both RGCs and neighboring retinal cells to elevated pressure, the use of retinal organ culture is essential. This approach offers significant advantages in preserving the anatomical structure and cell-to-cell interactions within retinal tissue during in vitro experiments. In contrast to studies using isolated RGCs, results from retinal organ cultures have demonstrated that elevated hydrostatic pressure (up to 90 mmHg) has no detectable effect on RGC survival [28,29,69]. The discrepancy between findings from these two types of models suggests that IOP-related RGC degeneration may be a multifactorial process involving not only elevated IOP but also the responses of other retinal cells and their interactions with RGCs. This further underscores the importance of utilizing retinal organ-based models in glaucoma research.

## 4. Development of Advanced In Vitro Microfluidic Models for Studying Degeneration of RGCs

While existing in vitro models, including static and dynamic pressure systems, have provided valuable insights into pressure-induced RGC degeneration, several limitations remain. These include poor standardization of pressure levels, limited physiological relevance in dynamic profiles (particularly in amplitude and frequency), and the oversimplification of cellular environments in monoculture settings. Furthermore, current models often fail to adequately incorporate the multicellular interactions and tissue-level context essential for understanding IOP-related RGC degeneration. To address these challenges, microfluidic technology offers promising opportunities to advance in vitro modeling.

Current microfluidic-based devices allow co-culturing of various types of cells, which facilitates the investigation of cell–cell interactions and replicates the structural stratification of multiple biological tissues [70,71,72,73,74,75,76,77]. Due to advancements in research on human induced pluripotent stem cells (hiPSCs), many microfluidic co-culture studies have replaced some primary cell types with hiPSC-derived substitutions, streamlining construction and enabling patient-specific disease modeling [78,79,80,81,82]. Stepping further, hiPSCs have been employed to derive 3D organoids that simulate in vivo tissue architectures, while limitations in reproducibility and long-term viability drive their integration with microfluidics [83,84]. The integrated platforms equip the organoids with dynamic flows for oxygen supply and nutrient/waste exchange, or even physiological micro-vascularization [85,86,87]. Enabled by 3D printing and photoresponsive hydrogels, synthetic vasculature-like microchannels can be created in organoids for precise control over flow dynamics and perfusion [88,89]. Merging microfluidics with organoids also offers advantages such as studying the collective activity of multiple organoids, creating physio-mimetic spatiotemporal chemical gradients to induce topographic differentiation, and high-throughput organoid imaging to examine drug toxicity and efficacy [90,91,92]. Finally, microfluidic platforms can compartmentalize the cultured subjects with microchannels [93,94,95,96,97,98]. This allows selective manipulation and analysis to precisely study the in vivo physically separated structures, such as neuronal soma and axons. All these strengths help improve the physiological relevance of in vitro studies, and further bridge their gap with animal research.

By applying the aforementioned microscale engineering technology, ocular tissue-like structures have been miniaturized, capturing key physiologically relevant microarchitectural features of the eye on small microfluidic chips [41]. For examples, multiple corneal layers can be recapitulated in a single microfluidic platform to replicate the stratified cornea and its barrier function [70,71,72,73,99]. Complex interactions between ocular surface tissues with external environmental factors can also be modeled with high fidelity [100,101]. A blinking human eye model was even interfaced with human-scale diagnostic tools and standard clinical tests on the ocular surface for studying dry eye diseases as well as conditions of the ocular surface [102]. Derived from hiPSCs, retinal organoids (ROs) comprise various major retinal cells and capture the retinal stratification. To further simulate in vivo retinal physiology, ROs have been merged with microfluidic technologies [103,104,105]. The integrated retina-on-a-chip recapitulates the interactions between photoreceptors with retinal pigment epithelium (RPE) [103]. It also provides vasculature-like perfusion and physio-mimetic oxygen gradients that vary across retinal layers, which enables the maturation of both inner and outer retinal cell phenotypes in ROs [104,105].

For highly relevant examples on glaucoma-related study, approaches include air-pressure systems integrated with polymethyl methacrylate (PMMA) chambers and fluid flow resistance in the microchannel [66,106]. A three-layered glaucoma-on-a-chip consisting of PMMA can be connected with an air-pressure source to elevate pressure inside the microfluidic chamber from 15 to 33 mmHg [66]. Employing air pressure facilitates more precise and stable control of gas flow velocity and composition in comparison to other in vitro models. In addition, by driving fluid with low flow rate through an agarose porous matrix in a microfluidic chamber, the static pressure elevation can also be generated and applied on the retina [106]. This approach mitigates the excessive shear stress exerted on tissues and cells in conventional microfluidic devices. Recent developments of microfluidics have also advanced the understanding of aqueous fluid built-up and resultant IOP elevation [107,108,109]. By modeling the aqueous outflow system consisting of the trabecular meshwork (TM) and Schlemm’s canal (SC), important characteristics of steroid-induced glaucoma can be recapitulated in vitro [109]. Altogether, these examples demonstrate the potential of the current engineering technologies in emulating the microenvironment of the eye, therefore offering new tools for enhancing our knowledge in the degeneration of RGCs within in vitro context. However, the etiology and mechanism of degeneration of RGCs, the related genetic analysis, and drug screening of potential drugs for RGCs should also be better emphasized [18].

In the following section, we outline key features for developing a next-generation in vitro model using microfluidic technologies to study pressure-induced RGC degeneration. Such a model should facilitate investigations ranging from isolated RGCs to multicellular co-culture systems, while enabling precise control over physiologically relevant parameters such as tissue deformation, dynamic IOP fluctuations, and intercellular biomechanical forces. We also discuss the potential of implementing microelectrode array into microfluidic platforms, which should enable precise detection of variation in the electrophysiological conductivity of RGCs, offering a new perspective to examine their electrophysiological responses under various mechanical stimuli.

### 4.1. Unidirectional Alignment of RGC Axons

**Figure 2:**
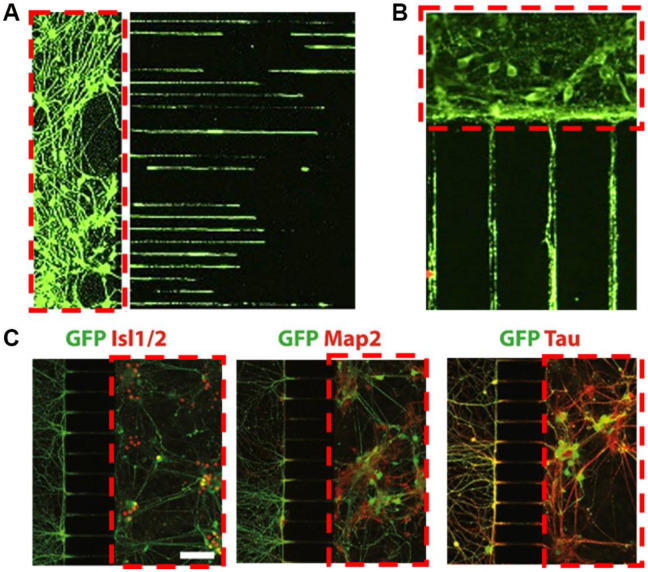
Representative images showing the varied spatial distributions and interconnections of neurons growing in microfluidic devices that separate soma and dendrites from axons of neural cells. Soma chambers are indicated by the red dotted rectangles. (**A**) Numerous cortical neurons from C57BL/6 mice are seeded in the soma chambers (**left**). Axons then grow along the microfluidic channels to the right [117]. (**B**) Cortical neurons from mice, with gene transfection, are cultured within the microfluidic device, allowing isolated neurites to elongate in microgrooves [118]. (**C**) Motor neurons differentiated from embryonic stem cell line (Hb9-GFP) are growing in the microfluidic chambers (**right**) and extending into the axonal compartment (**left**), with various immunostaining including GFP, Isl1/2, Map2, and Tau [116]. Scale bar: 500 μm. All the sub-figures in Figure 2 are under the terms of the Creative Commons CC-BY license.

Axons of RGCs converge to form the optic nerve, which in turn transmits visual information to the human brain. Since axon degeneration of RGCs is one of the key manifestations of glaucomatous patients, identifying the mechanism of axon degeneration can shed light on potential therapeutic strategies for glaucoma. While in vitro RGC studies have a variety of approaches in assessing the degeneration process [35,110,111], measurements of axon outgrowth [112] and axon degeneration index [113] are the more commonly used parameters to assess the degeneration of RGCs. However, regular neuronal cell culture methods are unable to separate axons from dendrites, hence they require additional steps of immunocytochemical staining to differentiate between these two cell types before performing axon length measurements. More recently, compartmentalized microfluidic chamber devices have been developed to separate neuronal soma and dendrites from axons [69,114]. The pressure difference, generated by the difference in height of cell culture medium between the two compartments in the microfluidic chamber device, guides the direction of axonal growth, ultimately providing a compartment containing only axons [114,115]. This design allows longitudinal follow-up of axon growth from the same dish of cultured RGCs without the need to perform immunocytochemical staining at various time points [112]. However, while such a microfluidic chamber can provide clear separation of RGC axons from somas and dendrites, the overall measured axon growth and axon degeneration index can also be influenced by the number of cells and their spatial distribution within the soma compartment, which may likely contribute to the low reproducibility between published in vitro studies (Figure 2) [116,117,118]. While microfluidic chambers offer an in vitro platform that separates RGC somas from axons to monitor the changes in axon growth, a chamber device that allows the monitoring of RGC axon growth at a single-cell level is not yet available.

To overcome this challenge, the latest micro-fabrication technologies may be applied to fabricate arrays of micro-cavities with precise dimensions that can provide the spatial resolution to capture single cells in individual cavities [119]. It is possible that individual cavities with an opening connection to the channels can be fabricated. Such design has the potential to be used to capture individual RGCs, allowing the monitoring of unidirectional axon growth of RGCs at a single-cell level, and in vitro investigations on the axon regenerative capacity of RGCs to be performed. Moreover, further development of such a device to a closed system may facilitate external application of hydrostatic pressure, expanding the platform for a wider range of single-cell investigations on pressure-induced degeneration of RGCs, such as longitudinal monitoring of changes in mitochondrial function. RGCs in glaucomatous eyes have impaired mitochondrial function [120], predisposing RGCs to apoptosis [121]. Understanding the relationship between the mitochondrial function of RGCs and pressure-induced degeneration of RGCs may allow for a better understanding of the disease progression of glaucoma from a biomechanical perspective.

### 4.2. Co-Culture Systems That Allow Cell–Cell Interactions Between Retinal Cell Types

Cell co-culturing models are fundamental tools to investigate the role of cell–cell communication in degeneration. Co-culture in vitro models, comprising two or more different types of cells, attempt to simulate the surrounding environment found in tissues, thus providing a different cellular composition in vitro compared with single-cell models [26,122,123,124]. Co-culture of various retinal cell types (such as retinal ganglion cells, bipolar cells, glial cells, and photoreceptors) can be facilitated using microfluidic platforms featuring separated yet interconnected parallel channels or chambers. These compartments are linked via microchannels, allowing controlled intercellular communication while preserving essential biological characteristics, including barrier integrity, retinal-specific gene expression, and light sensitivity [125]. Under well-defined pressure levels and physiologically relevant micro-environments, such configuration facilitates investigations on neuronal development of RGCs, intercellular communication, and the role of paracrine signaling in neurodegeneration [114]. Specifically, the blockade of microglial antagonists of the adenosine A2A receptor prevents microglial cell response to elevated pressure, thereby potentially protecting RGCs from elevated pressure-induced death [126]. This further highlights the importance of cell–cell interactions between retinal cell types when studying pressure-induced degeneration of RGCs in vitro. Another advanced approach involves retina-on-a-chip models, which leverage microfluidic organ-on-a-chip technology to replicate not only multicellular interactions but also higher-order tissue-level functions [127]. While the fundamental principles of these systems have been established, further research is needed to incorporate the full complexity of retinal architecture and physiology before they can be reliably applied in disease modeling and therapeutic development [127].

### 4.3. The Control of Various Biomechanical Parameters That RGCs Experience In Vivo

Cells inside the human body are continuously exposed to biomechanical stimuli [128]. Microfluidic in vitro platforms have the advantage of providing precise and controllable biophysical cues, such as fluid shear stress, mechanical stretch, and compression [129]. This advantage enables more physiologically relevant studies on the degeneration of RGCs, in terms of the dynamic change in IOP and the deformation of scleral tissue under fluctuating IOP. Previous studies indicated that optic neuropathy might result from the deformation of load-bearing tissues of the optic nerve head (ONH) [130,131]. The laminar region of ONH, also known as lamina cribrosa (LC), is a trabecular structure across which RGC axons leave the eye on their path to the brain. However, due to its low-loading-bearing tissue components and thin structure, LC deforms the most within ONH under elevated IOP [132]. Such deformation induce mechanical stresses on, and thus mechanically damages, the RGCs axons that pass through lamina cribrosa [133]. The effect of elevated IOP on the ONH and LC has also been explored using computational modeling [134]. However, these models could not examine the direct effect of IOP changes on individual RGCs.

#### 4.3.1. Tissue Biomechanics

Precise manipulation of biophysical stimulation inside microfluidic devices facilitates mechanobiological studies on pressure-induced degeneration of RGCs. For example, the substrate that RGC grows on can be tuned to match the biomechanical properties of the lamina cribrosa. The stiffness of lamina cribrosa, measured by atomic force microscope (AFM), lies between 16.7 (±4.8) and 17.7 (±2.8) kPa [135], depending on the exact anatomical location. Polydimethylsiloxane (PDMS), a transparent material that is commonly used to make microfluidic chips for in vitro cell culture studies, can be altered slightly to simulate the mechanical properties of lamina cribrosa by adjusting the ratio between the PDMS pre-polymer and the curing agent. The thickness of the walls of the PDMS-based device can also be customized to match the thickness of human lamina cribrosa. Therefore, the deformation of a PDMS wall under different pressures becomes possible to mimic the physical distortion of the optic nerve head induced by IOP [130,131].

#### 4.3.2. Fluctuating IOP Levels

Elevation of IOP is a major risk factor for RGC degeneration in glaucoma. However, IOP fluctuates with both the circadian rhythm [136] and cardiac rhythm [137]. Whether pressure fluctuation plays a significant role similar to IOP elevation in RGC degeneration remains elusive [5]. The difference in diurnal and nocturnal IOP can be as high as 5.0 ± 0.3 mmHg in healthy subjects, but less substantial in early glaucoma patients (2.7 ± 0.3 mmHg). Meanwhile, the ocular pulse amplitude (OPA), defined as the difference between systolic and diastolic IOP, ranges between 0.9 and 7.2 mmHg in healthy subjects, with a mean of 2.58 mmHg [56,138]. However, the OPA in glaucoma patients could vary from 0.7 to 8.9 mmHg, with the mean of 2.85 mmHg [139]. Recent studies have suggested that OPA may also be a key factor for the development of glaucoma [54,55]. Cyclic mechanical stress can be more harmful to neurons than constant stress as supported by both in vitro and in vivo evidence [140]. Furthermore, the majority of prior in vitro pressure models lack comprehensive oxygen monitoring, which consequently impedes the validation of device biocompatibility. Future developments in dynamic IOP modeling should incorporate appropriately oxygenated microenvironments to ensure that any observed cellular degeneration is not erroneously attributed to hypoxia. Therefore, the correct simulation of dynamic pressure profiles within biocompatible microfluidic devices is essential to simulate the clinical physiological IOP profiles [35,128]. This would facilitate investigations into how amplitude of fluctuating IOP plays a role in pressure-induced RGC degeneration.

#### 4.3.3. Mechanical Stresses Acting on RGC Axons Due to the Deformation of Laminar Cribrosa

**Figure 3:**
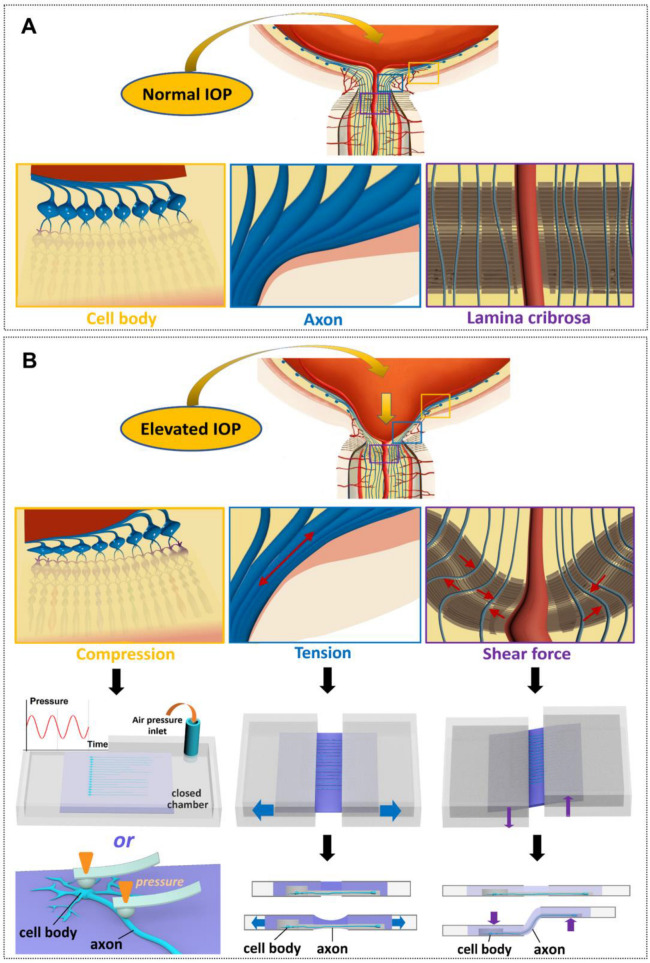
Schematics showing the types of biomechanical stimuli acting on RGCs at different regions within the eye. (**A**) Schematic showing the RGCs at various regions within the eye under normal IOP. (**B**) Elevated IOP directly induces compression, tension and shear forces on RGCs at various regions within the eye, which can be modeled using in vitro flexible microfluidic platforms.

Optic disc excavation is the characteristic feature of IOP elevation, which corresponds to the deformation of optic nerve head (ONH) (Figure 3A). Under such deformation, various stresses and strains are induced and applied directly on the RGC axons near ONH. As described earlier, LC, the softest region in ONH, deforms the most. These stresses may also be important biomechanical parameters to explain pressure-induced degeneration of RGCs [141,142,143]. ONH can be displaced by as much as around 100 µm with a baseline IOP of 30 mmHg [144], and RGC axons around ONH will experience a tensile force causing them to elongate. The deformation of LC under IOP elevation can also exert shear stress directly on the RGC axons that pass through LC (Figure 3B). With the use of tailored-made microfluidic platforms incorporating other instrumentations such as uniaxial stretching machines, repetitive tensile and shear stresses may be applied directly on RGCs that are cultured in flexible microfluidic devices that deform according to the applied stress (Figure 3B). Based on this concept, the flexible microchannels that allow the axonal growth should be made softer to mimic LC region among scleral tissue. Accordingly, the microchannels will deform the most under the concentration of applied stress, allowing repetitive tension and strains on the RGC axons. Applying and retracting repetitive stresses directly on RGC neurons can help us to understand the potential role of material fatigue. If possible, this type of prototype of LC deformation modeling will contribute to study towards pressure-induced degeneration of RGCs from a biomechanical perspective, potentially paving a way for IOP management to better prevent glaucoma.

### 4.4. Merging Microfluidics with Electrode Technology to Study RGC Electroconductivity

To further investigate the physiological responses of RGC under mechanical stimulation, microelectrodes can be integrated into in vitro microfluidic platforms to probe its electrophysiological conductivity [145], which offers quantitative evaluations of the cellular physiological function. Such microelectrode devices have been developed to investigate the electrophysiology of in vitro single neuronal cells such as hippocampal neurons, as well as ex vivo retinas [146,147,148], thereby providing significant potential for application in studying single RGCs. Although microelectrodes have been employed to stimulate and record signals from RGC populations [149,150,151,152], these studies examined collective profiles without resolving electrophysiological properties of an individual RGC. To combat this limitation, an in vitro single-RGC microfluidic apparatus equipped with microelectrodes should be constructed [147,153,154]. Such an approach would enable unambiguous detection of alternations in RGC electroconductivity under mechanical stimulation, offering new insights into mechanisms of RGC degeneration from biomechanical perspectives.

Functionally, the single-RGC microelectrode platforms should consist of three essential parts, namely, stimulation of RGC to elicit action potential, signal recording at axon initial segment (AIS), and multisite recording along axon length. AIS is a specialized region at the proximal end of the axon adjacent to the cell body. It accommodates voltage-gated ion channels clustered at high density, acting as the site of action potential initiation preceding its propagation along the axon [155,156,157,158]. By processing the bioelectrical signals acquired at AIS and along axon length, RGC electroconductivity can be investigated.

**Figure 4:**
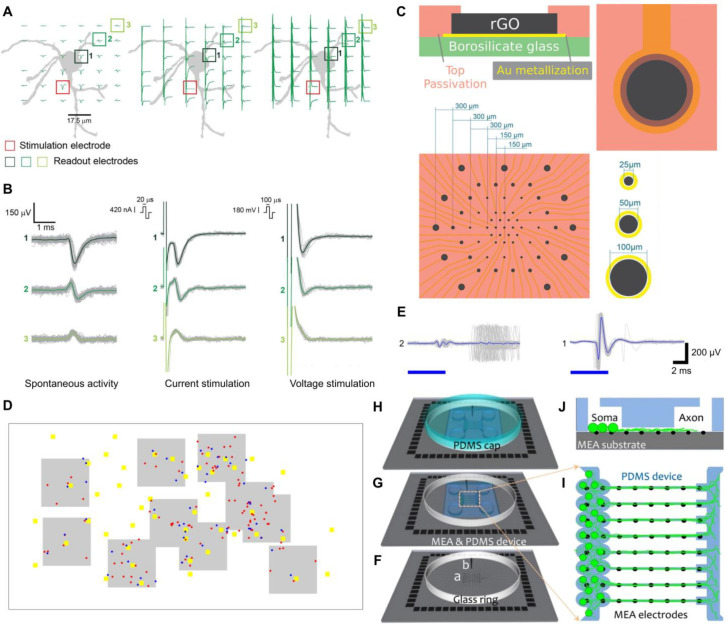
Microelectrode platforms to be translated to study single RGC. (**A**) Positions of electrodes with overlaid recorded signals. Left: spontaneous extracellular action potentials (EAPs). Middle: EAPs after current stimulation. Right: EAPs after voltage stimulation [146]. (**B**) Signals from the readout electrodes in (**A**) that are indicated by the numbered box 1, 2, 3. Left: extracellular signals captured during spontaneous neuronal activity. Middle and Right: extracellular signals captured during current and voltage stimulation [146]. (**C**) Upper panel: cross-section and top view of one rGO microelectrode. Lower panel: array design of various electrode sizes and pitches [148]. Compared to traditional metal-based MEAs, rGO MEAs boast the advantages of low impedance and high charge injection limits, enhancing stimulation and recording efficiency [148,164]. (**D**) Gray shades: HD-MEA recording areas. Yellow squares: optical stimulation sites. Blue and red dots: directly and indirectly responding individual neurons. Directly responding neurons are those targeted by optical stimulation, which transmit electrical signals via synaptic connections and activate indirectly responding neurons [161]. (**E**) Signals recorded by two widely separated electrodes, 1 and 2. 1: direct responses. 2: indirect responses. The blue bars indicate stimulation period. Gray: signal at each stimulus. Blue: stimulus-time-triggered averaged signals [161]. (**F**) MEA on a substrate, (a) readout electrodes and (b) counter electrode. A glass ring for confining culture medium. (**G**) A PDMS layer attached onto the substrate to create microwell and microchannel geometries. Cells are seeded through the four circular openings. (**H**) A PDMS cap sealing the culture. (**I**,**J**) Cross-section and top view of internal architectures. Five electrodes monitor axons along each channel [163]. All the sub-figures in Figure 4 are under the terms of the Creative Commons CC-BY license http://creativecommons.org/licenses/by/4.0/ (accessed on 23 September 2025).

To realize electrical stimulation to a RGC while recording its responses near the cell body and AIS, multielectrode arrays (MEA) prove to be a promising approach. They allow bi-directional interfacing with neurons and have been utilized to probe neuronal activities in several studies [159,160]. High-density microelectrode arrays (HD-MEA) achieved focal electrical stimuli to the AIS to initiate the action potential of a single cortical neuron, followed by recording responses near the cell body and AIS on the same HD-MEA device (Figure 4A,B) [146]. Another platform using reduced graphene oxide (rGO) MEA enabled real-time modulation and monitoring of neuronal activities of hippocampal neurons and ex vivo retinas (Figure 4C) [148]. Nevertheless, the use of these purely electrical methods to capture physiological signals from neuronal cells and tissues faces challenges, such as the presence of artifacts interfering with the recording of neuronal activities immediately after stimulation [161]. To address this issue, a digital micromirror device (DMD) was employed to generate patterned light stimuli, triggering electrical responses in rat cortical neurons captured by electrodes [161]. The optical stimulation and HD-MEA recording were both on a single-neuron level (Figure 4D,E).

Lastly, to perform multisite recording along the axon of a single RGC, a sandwich configuration can be constructed by overlaying a PDMS film onto a planar MEA [162,163]. The PDMS film typically includes microwells and microchannels to confine cell bodies and guide axons extending across the electrode array, respectively. Neurites of a snail neuron were guided to grow from the soma chamber, passing a set of parallel indium tin oxide (ITO) electrodes along a linear microchannel to record electrical activities at different segments [162]. Similar principles were applied to rat cortical neurons (Figure 4F–J) [163], implying the potential of these platforms in recording electrical signals from individual RGCs across a range of spatial regions.

## 5. Conclusions and Future Perspectives

Glaucoma is a neurodegenerative disorder with complicated pathogenesis that is far from being completely understood. While in vivo studies are vital for understanding disease mechanisms and developing therapeutic strategies, precise IOP regulation is challenging for both animal models and clinical settings. Microfluidic technologies have been widely applied to facilitate biomedical research over the years, but their use in vision research remains limited. The combination of current microfluidic technologies and RGC culture may advance in vitro platforms to monitor and detect changes in RGCs under the mechanical pressure. In vitro microfluidic systems enable regulatable pressure-related parameters including pressure type (static/dynamic), intensity, and frequency, which are useful for providing complementary assessments that require precise control of IOP. Moreover, the precise parameters of microfluidic design could be achieved, such as elasticity of soft materials, biomechanical stimuli, individual cavities for single-cell capture, and customizable compartmentalization for co-cultures. These advantages contribute to the high physiological relevance of the in vitro platform, thus bridging the research gap between conventional in vitro RGC culture and animal studies. Real-time monitoring of intracellular changes of RGCs under desired pressure will be useful to improve our understanding of the effect of increased pressure at a single-cell level. Moreover, this may aid single-cell investigations on pressure-induced changes in intracellular mechanisms, particularly transcriptomic, proteomic, and metabolic features. Various force spectroscopy technologies, including AFM, optical tweezers, magnetic tweezers, and traction force microscopy, can help correlate the mitochondrial function of RGCs with biomechanics. Collectively, microfluidic systems provide a new paradigm to explore the association between IOP fluctuations and the degeneration of RGCs, potentially deepening our understanding of the pathogenesis of IOP-induced glaucoma. Future integration of microfluidic technologies in vision research may further maximize opportunities to study various other ocular diseases.

## Figures and Tables

**Table 1:** The in vitro pressurized systems employed to investigate pressure-induced degeneration of RGCs through direct application of pressure to the cells.

Reference	Cell Type(s) Involved	Study Targets	System and Experimental Design	Main Findings	Conclusion/Significance	Limitations
Ju et al., 2007 [27]	RGC-5 cell line RGC-5 cells were differentiated with succinyl concanavalin A	Mitochondrial dysfunction in RGC-5	Pressure type: Hydrostatic Setup: Pressurized Chamber Duration: 3 days Pressure level (mmHg): 30	- Elevated hydrostatic pressure triggered mitochondrial fission, abnormal cristae depletion, and Drp-1 translocation. - ↓ Cellular ATP and ↓ Cell survival after 3 days	- Regulating the cellular response to elevated pressure including mitochondrial fission may provide new therapeutic targets for protecting RGCs from elevated pressure.	- RGC-5 cell line has been abandoned in vision science research. - The mechanism of cellular effects due to elevated pressure is still unclear.
Liu et al., 2007 [28]	RGC-5 cell line	Oxidative adduct formation and heme oxygenase-1 (HO-1) expression in RGC-5	Pressure type: Hydrostatic Setup: Pressurized Chamber Duration: 2 h Pressure level (mmHg): 30, 60 and 100	- ↑ hydroxy-2-nonenal (HNE)-protein adducts and ↑ HO-1 expression - During recovery experiments, ↑ HNE-protein adducts but ↓ HO-1 expression - HNE, led to neurotoxicity in RGC-5 cells. - Antioxidant treatment reduced the oxidative stress level in pressure-treated RGC-5 cells	- Oxidative stress is anearly event in hydrostatic pressure/IOP-induced neuronal damage.	- RGC-5 cell line has been abandoned in vision science research. - The study cannot prove why neuronal cell loss only localises at RGCs but not other retinal neurons.
Resta et al., 2007 [39]	Rat RGCs in isolated rat retinas	Viability of RGCs	Pressure type: Dynamic (Pulsatile) Setup: Pressurized Chamber (regulated by electronically controlled gas admission) Duration: 1 h Pressure level (mmHg): 50	- Slowly rising pressure to peaks did not damage RGCs, whereas a rapid 1 min-pulse to 50 mmHg injured 30% of RGCs within 1 h. - The severity of damage and the number of affected cells increased with stronger or repeated insults. - Degrading extracellular ATP or blocking the P2X receptors for ATP prevented acute pressure-induced damage in RGCs. - A short intraocular pressure transient increased extracellular ATP levels in the eye fluids and damaged RGCs within 1 h. - Reducing extracellular ATP in the eye prevented damage to RGCs and accelerated recovery of their response to light.	- The rapid pressure transients induce acute RGC injury and unveils the causal role of extracellular ATP elevation in such injury.	- Only when the pressure is applied quickly did it have an effect on RGC viability. An increase of ~3 mmHg did not cause loss of viability, even if the pressure is increased finally to 50 mmHg. - Cannot determine if age of RGCs is a potential confounder.
Sappington et al., 2009 [33]	Primary mice RGCs	The transient receptor potential vanilloid 1 (TRPV1) channel	Pressure type: Hydrostatic Setup: Pressurized Chamber Duration: 48 h Pressure level (mmHg): 70	- TRPV1 antagonism increased cell density and reduced apoptosis to ambient levels, whereas for RGCs at ambient pressure, TRPV1 agonism reduced density and increased apoptosis to levels for elevated pressure. - Chelation of extracellular Ca^2+^ reduced RGC apoptosis at elevated pressure by nearly two-fold. Exposure to elevated hydrostatic pressure induced a four-fold increase in RGC intracellular Ca^2+^ that was reduced by half with TRPV1 antagonism.	- RGC apoptosis induced by elevated hydrostatic pressure arises substantially through TRPV1, likely through the influx of extracellular Ca^2+^.	- TRPV1 in RGCs can be activated by pressure and contributes to a pressure-dependent signal, the study did not prove whether this activation arised from static pressure or a pressure gradient, or an aqueous gradient arising from increased pressure.
Osborne et al., 2015 [40]	Primary RGCs in human reina explant Human organotypic retinal cultures (HORCs) from donor eyes were cultured in serum-free DMEM/hamf12.	Stress pathway signaling and RGCs survival	Pressure type: Both Hydrostatic and Dynamic (1/60 Hz, 10–100 mmHg) Setup: Pressurized Chamber with mass flow controllers Duration: 24 h (static pressure)/1 h cyclic pressure Pressure level (mmHg): 60	- No loss of structural integrity, increase in TUNEL-positive neuon-labelled cells LDH release, decrease in RGC marker expression or loss of RGCs	- No detectable impact on RGC survival and stress-signalling in horcs.	- The use of organotypic retinal culture instead of microglial culture may mean that glial cells other than microglia may have an additional contribution to A2AR activity. - Cellular source of extracellular ATP is unclear.
Wu et al., 2019 [35]	Primary rats RGCs	Biological changes of RGCs (axon and total neurite length, cell body area, dendritic branching, and cell survival)	Pressure type: Hydrostatic Setup: Liquid height Duration: 72 h Pressure level (mmHg): 10, 20, 25, 30, 40 and 50	- Pressure-dependent changes in the axon length, and the total neurite length were shown. - RGCs degenerate at pressures above 25 mmHg. - No significant change in cell body area under different levels of hydrostatic pressure was observed.	- There is a critical pressure threshold at approximately 25 mmHg, beyond which RGCs become vulnerable to cell death, which is comparable to clinical findings.	- Did not obtain oxygen tension measurements on this model so it is unclear if hypoxia is a confounding factor in this system.
Nafian et al., 2020 [66]	Primary rat RGCs purified from postnatal Wistar rats	Potential effect of neuroprotection of brain-derived neurotrophic factor (BDNF) or a novel BDNF mimetic (RNYK) on RGCs	Pressure type: Hydrostatic Setup: Pressurized chamber Duration: 6, 12, 24, 36, and 48 h Pressure level (mmHg): 15 and 33	- RGC survival rates were significantly higher under normal pressure (15 mmHg) then under high pressure (33 mmHg) at all time points. - BDNF and RNYK separately reduced RGC death rates about two-fold under both normal and elevated pressures.	- This model recapitulated the effects of elevated pressure over relatively short time periods and demonstrated the neuroprotective effects of BDNF and RNYK.	- Focuse is on the development of the chip system rather than the studying of effect of RGC under high pressure. - The 3-layered chip design gives inappropriate surface tension leading to uneven distribution of the culture media.

**Table 2:** The in vitro studies related to responses of other retinal cells to elevated pressures.

Reference	Target Cell Type(s)	Study Aims	Experimental Design	Main Findings	Conclusion/Significance	Limitations
Agar et al., 2000 [34]	Neuronal cell lines (B35 and PC12) B35 line is derived from the CNS of rat.	Cultured neuronal lines	Pressure type: Hydrostatic Setup: Pressurized Chamber Duration: 2 h Pressure level (mmHg): 100	- ↑ apoptosis (by TUNEL and Annexin V) at 2 h, but not 6 or 20 h.	Pressure alone may act as a stimulus for apoptosis in neuronal cell cultures.	- Apoptosis is used as the outcome variable, which may be less reliable since cells may be at different stages of apoptosis. - The underlying mechanism is unclear since apoptosis is the outcome studied.
Sappington et al., 2006 [32]	Primary rat RGCs, astrocyte, and microglia	Glia-derived factors, in particular interleukin-6 (IL-6), on RGC survival	Pressure type: Hydrostatic Setup: Pressurized Chamber Duration:0–48 h Pressure level (mmHg): 30, 70	- Pressure elevated for 24 to 48 h ↓ density, ↑ TUNEL labeling, ↑ apoptotic genes, including c-jun and jun-B of RGCs. - Pressure-conditioned medium from astrocytes reduced RGCs survival, while microglia medium returned RGC survival to ambient levels. These effects were unrelated to IL-6 in microglia medium. - Neither astrocyte- nor microglia-conditioned medium affected ambient RGCs survival unless depleted of IL-6. Recombinant IL-6 equivalent to levels in glia-conditioned medium doubled RGC survival at elevated pressure.	Increased IL-6 in microglia medium counters not only proapoptotic signals from these cells but also the pressure-induced apoptotic cascade intrinsic to RGCs.	- Further investigations should examine the impact of IL-6 signaling in vivo in which responses can differ from those in vitro.
Mandal et al., 2010 [43]	Primary rat optic nerve astrocytes	Realtionship between calcium responses and ERK1/2 phosphorylation.	Pressure type: Hydrostatic Setup: Liquid height Duration: 2 h Pressure level (mmHg): 15	- Elevated pressure caused ERK1/2 phosphorylation. The magnitude of the ERK1/2 phosphorylation response was reduced by ruthenium red and dantrolene.	- Increasing preesure causes calcium release from a ryanodine-sensitive cytoplasmic store and subsequent ERK1/2 activation. - Calcium store release appears to be a required early step in the initial astrocyte response to a pressure increase.	- Mechanism of morphologocal changes and increased beading in glaucomatous axons remains unclear.
Yu et al., 2011 [38]	Rat retinal Müller cells	Glutamine synthetase (GS) in rat retinal Müller cells	Pressure type: Hydrostatic Setup: Pressurized cell culture flask Duration: 24 h Pressure level (mmHg): 20, 40, 60, 80	- ↑ expression of GS and GS mRNA expression in both the 40 mmHg/24 h and the 60 mmHg/24 h groups	- Müller cells adaptively upregulate GS in an elevated hydrostatic pressure to degrade glutamate, which may lead to neuronal damage at high concentrations	- Unable to rule out if there are any mechanical effect exerted by the pressure-induced coverslip deformation of the experiment. - Source of ATP cannot be pinpointed since it may be released from multiple sources.
Lei et al., 2011 [29]	DITNC1 cell line, a kind of optic nerve head [ONH] cells DITNC1 cells are obtained from brain diencephalon tissues of 1-day-old rats.	Potential biological changes of ONH cells cultured on a rigid substrate (migration, shape, and α-tubulin architecture in the cells)	Pressure type: Hydrostatic Setup: Liquid height Duration: 48/72 h Pressure level (mmHg): 7.4	- No effects of hydrostatic pressure were observed on cell migration or α-tubulin architecture. - Cells cultured under low gas tension showed significant increased migration at 48 and 72 h.	- Gas tension rather than hydrostatic pressure affects cell migration	- Hydrostatic pressure is difficiult to be changed without altering the gas tension in culture medium. - The elevated pressure value (7.4 mmHg) was not high enough to mimic the physiological elevation of IOP.
Madeira et al., 2015 [30]	Microglia and RGCs in rat retinal organotypic cultures	The ability of A2AR blockade to control the reactivity of microglia and neuroinflammation as well as RGC loss in retinal organotypic cultures	Pressure type: Hydrostatic Setup: Pressurized Chamber Duration: 4/24 h Pressure level (mmHg): 70	- ↑ expression of A2AR in retinal microglial cells, and altered microglia to a more amoeboid-like morphology. ↑ mRNA expression of iNOS, ↑ NO production and ↑ IL-1β. - The A2AR antagonist (SCH 58261) prevented microglia reactivity Neutralization of TNF and IL-1β prevented RGC loss	- The triggered microglial reactivity contributes to the pathophysiology of glaucoma by impairment of RGC viability. - A2AR blockade confers neuroprotection to RGCs by controlling microglia-mediated retinal neuroinflammation.	- Unable to explain the increase in glutamate during elevated hydrostatic pressure.

## Data Availability

No new data were created or analyzed in this study.

## References

[1] Tham Y.-C., Li X., Wong T.Y., Quigley H.A., Aung T., Cheng C.-Y. (2014). Global Prevalence of Glaucoma and Projections of Glaucoma Burden through 2040: A Systematic Review and Meta-Analysis. Ophthalmology.

[2] Bouhenni R.A., Dunmire J., Sewell A., Edward D.P. (2012). Animal Models of Glaucoma. J. Biomed. Biotechnol..

[3] Morrison J.C., Moore C.G., Deppmeier L.M.H., Gold B.G., Meshul C.K., Johnson E.C. (1997). A Rat Model of Chronic Pressure-induced Optic Nerve Damage. Exp. Eye Res..

[4] Johnson E.C., Morrison J.C., Farrell S., Deppmeier L., Moore C.G., McGinty M.R. (1996). The Effect of Chronically Elevated Intraocular Pressure on the Rat Optic Nerve Head Extracellular Matrix. Exp. Eye Res..

[5] Casson R.J., Chidlow G., Wood J.P.M., Crowston J.G., Goldberg I. (2012). Definition of glaucoma: Clinical and experimental concepts. Clin. Exp. Ophthalmol..

[6] Goel M., Picciani R.G., Lee R.K., Bhattacharya S.K. (2010). Aqueous humor dynamics: A review. Open Ophthalmol. J..

[7] Brubaker R.F. (1991). Flow of aqueous humor in humans [The Friedenwald Lecture]. Investig. Ophthalmol. Vis. Sci..

[8] Urcola J.H., Hernández M., Vecino E. (2006). Three experimental glaucoma models in rats: Comparison of the effects of intraocular pressure elevation on retinal ganglion cell size and death. Exp. Eye Res..

[9] Biermann J., van Oterendorp C., Stoykow C., Volz C., Jehle T., Boehringer D., Lagrèze W.A. (2012). Evaluation of intraocular pressure elevation in a modified laser-induced glaucoma rat model. Exp. Eye Res..

[10] Johnson B., House P., Morgan W., Sun X., Yu D.-Y. (1999). Developing laser-induced glaucoma in rabbits. Aust. N. Z. J. Ophthalmol..

[11] Strouthidis N.G., Fortune B., Yang H., Sigal I.A., Burgoyne C.F. (2011). Effect of Acute Intraocular Pressure Elevation on the Monkey Optic Nerve Head as Detected by Spectral Domain Optical Coherence Tomography. Investig. Ophthalmol. Vis. Sci..

[12] Aihara M., Lindsey J.D., Weinreb R.N. (2003). Ocular Hypertension in Mice with a Targeted Type I Collagen Mutation. Investig. Ophthalmol. Vis. Sci..

[13] Skarie J.M., Link B.A. (2008). The Primary open-angle glaucoma gene WDR36 functions in ribosomal RNA processing and interacts with the p53 stress–response pathway. Hum. Mol. Genet..

[14] Toris C.B., Zhan G.-L., Wang Y.-L., Zhao J., McLaughlin M.A., Camras C.B., Yablonski M.E. (2000). Aqueous Humor Dynamics in Monkeys with Laser-Induced Glaucoma. J. Ocul. Pharmacol. Ther..

[15] Harada T., Harada C., Nakamura K., Quah H.-M.A., Okumura A., Namekata K., Saeki T., Aihara M., Yoshida H., Mitani A. (2007). The potential role of glutamate transporters in the pathogenesis of normal tension glaucoma. J. Clin. Investig..

[16] Whitesides G.M. (2006). The origins and the future of microfluidics. Nature.

[17] Huh D., Matthews B.D., Mammoto A., Montoya-Zavala M., Hsin H.Y., Ingber D.E. (2010). Reconstituting Organ-Level Lung Functions on a Chip. Science.

[18] El-Ali J., Sorger P.K., Jensen K.F. (2006). Cells on chips. Nature.

[19] Leske M.C., Heijl A., Hussein M., Bengtsson B., Hyman L., Komaroff E. (2003). Factors for glaucoma progression and the effect of treatment: The early manifest glaucoma trial. Arch. Ophthalmol..

[20] Chan K.C., Yu Y., Ng S.H., Mak H.K., Yip Y.W.Y., van der Merwe Y., Ren T., Yung J.S.Y., Biswas S., Cao X. (2019). Intracameral injection of a chemically cross-linked hydrogel to study chronic neurodegeneration in glaucoma. Acta Biomater..

[21] Mansouri K., Tanna A.P., De Moraes C.G., Camp A.S., Weinreb R.N. (2020). Review of the measurement and management of 24-hour intraocular pressure in patients with glaucoma. Surv. Ophthalmol..

[22] Mansouri K., Weinreb R.N., Medeiros F.A. (2013). Is 24-hour intraocular pressure monitoring necessary in glaucoma?. Semin. Ophthalmol..

[23] Leidl M.C., Choi C.J., Syed Z.A., Melki S.A. (2014). Intraocular pressure fluctuation and glaucoma progression: What do we know?. Br. J. Ophthalmol..

[24] Esch E.W., Bahinski A., Huh D. (2015). Organs-on-chips at the frontiers of drug discovery. Nat. Rev. Drug Discov..

[25] Bhatia S.N., Ingber D.E. (2014). Microfluidic organs-on-chips. Nat. Biotechnol..

[26] Wang L., Tao T., Su W., Yu H., Yu Y., Qin J. (2017). A disease model of diabetic nephropathy in a glomerulus-on-a-chip microdevice. Lab Chip.

[27] Ju W.-K., Liu Q., Kim K.-Y., Crowston J.G., Lindsey J.D., Agarwal N., Ellisman M.H., Perkins G.A., Weinreb R.N. (2007). Elevated Hydrostatic Pressure Triggers Mitochondrial Fission and Decreases Cellular ATP in Differentiated RGC-5 Cells. Investig. Ophthalmol. Vis. Sci..

[28] Liu Q., Ju W.-K., Crowston J.G., Xie F., Perry G., Smith M.A., Lindsey J.D., Weinreb R.N. (2007). Oxidative Stress Is an Early Event in Hydrostatic Pressure–Induced Retinal Ganglion Cell Damage. Investig. Ophthalmol. Vis. Sci..

[29] Lei Y., Rajabi S., Pedrigi R.M., Overby D.R., Read A.T., Ethier C.R. (2011). In Vitro Models for Glaucoma Research: Effects of Hydrostatic Pressure. Investig. Ophthalmol. Vis. Sci..

[30] Madeira M.H., Elvas F., Boia R., Gonçalves F.Q., Cunha R.A., Ambrósio A.F., Santiago A.R. (2015). Adenosine A2AR blockade prevents neuroinflammation-induced death of retinal ganglion cells caused by elevated pressure. J. Neuroinflamm..

[31] Sumpio B.E., Widmann M.D., Ricotta J., Awolesi M.A., Watase M. (1994). Increased ambient pressure stimulates proliferation and morphologic changes in cultured endothelial cells. J. Cell. Physiol..

[32] Sappington R.M., Chan M., Calkins D.J. (2006). Interleukin-6 Protects Retinal Ganglion Cells from Pressure-Induced Death. Investig. Ophthalmol. Vis. Sci..

[33] Sappington R.M., Sidorova T., Long D.J., Calkins D.J. (2009). TRPV1: Contribution to retinal ganglion cell apoptosis and increased intracellular Ca2+ with exposure to hydrostatic pressure. Investig. Ophthalmol. Vis. Sci..

[34] Agar A., Yip S.S., Hill M.A., Coroneo M.T. (2000). Pressure related apoptosis in neuronal cell lines. J. Neurosci. Res..

[35] Wu J., Mak H.K., Chan Y.K., Lin C., Kong C., Leung C.K.S., Shum H.C. (2019). An in vitro pressure model towards studying the response of primary retinal ganglion cells to elevated hydrostatic pressures. Sci. Rep..

[36] Ingensiep C., Schaffrath K., Walter P., Johnen S. (2022). Effects of Hydrostatic Pressure on Electrical Retinal Activity in a Multielectrode Array-Based ex vivo Glaucoma Acute Model. Front. Neurosci..

[37] Kashiwagi K., Iizuka Y., Tanaka Y., Araie M., Suzuki Y., Tsukahara S. (2004). Molecular and Cellular Reactions of Retinal Ganglion Cells and Retinal Glial Cells under Centrifugal Force Loading. Investig. Ophthalmol. Vis. Sci..

[38] Yu J., Zhong Y., Cheng Y., Shen X., Wang J., Wei Y. (2011). Effect of high hydrostatic pressure on the expression of glutamine synthetase in rat retinal Müller cells cultured in vitro. Exp. Ther. Med..

[39] Resta V., Novelli E., Vozzi G., Scarpa C., Caleo M., Ahluwalia A., Solini A., Santini E., Parisi V., Di Virgilio F. (2007). Acute retinal ganglion cell injury caused by intraocular pressure spikes is mediated by endogenous extracellular ATP. Eur. J. Neurosci..

[40] Osborne A., Aldarwesh A., Rhodes J.D., Broadway D.C., Everitt C., Sanderson J. (2015). Hydrostatic pressure does not cause detectable changes in survival of human retinal ganglion cells. PLoS ONE.

[41] Zheng F., Fu F., Cheng Y., Wang C., Zhao Y., Gu Z. (2016). Organ-on-a-Chip Systems: Microengineering to Biomimic Living Systems. Small.

[42] Sappington R.M., Calkins D.J. (2006). Pressure-Induced Regulation of IL-6 in Retinal Glial Cells: Involvement of the Ubiquitin/Proteasome Pathway and NFκB. Investig. Ophthalmol. Vis. Sci..

[43] Mandal A., Shahidullah M., Delamere N.A. (2010). Hydrostatic pressure-induced release of stored calcium in cultured rat optic nerve head astrocytes. Investig. Ophthalmol. Vis. Sci..

[44] Hui T.H., Zhou Z.L., Qian J., Lin Y., Ngan A.H.W., Gao H. (2014). Volumetric Deformation of Live Cells Induced by Pressure-Activated Cross-Membrane Ion Transport. Phys. Rev. Lett..

[45] Ishikawa M., Yoshitomi T., Zorumski C.F., Izumi Y. (2010). Effects of acutely elevated hydrostatic pressure in a rat ex vivo retinal preparation. Investig. Ophthalmol. Vis. Sci..

[46] Ishikawa M., Yoshitomi T., Covey D.F., Zorumski C.F., Izumi Y. (2016). TSPO activation modulates the effects of high pressure in a rat ex vivo glaucoma model. Neuropharmacology.

[47] Cohen L.P., Pasquale L.R. (2014). Clinical characteristics and current treatment of glaucoma. Cold Spring Harb. Perspect. Med..

[48] Silver D.M., Farrell R.A., Langham M.E., O’Brien V., Schilder P. (1989). Estimation of pulsatile ocular blood flow from intraocular pressure. Acta Ophthalmol. Suppl..

[49] Zion I.B., Harris A., Siesky B., Shulman S., McCranor L., Garzozi H.J. (2007). Pulsatile ocular blood flow: Relationship with flow velocities in vessels supplying the retina and choroid. Br. J. Ophthalmol..

[50] Findl O., Rainer G., Dallinger S., Dorner G., Polak K., Kiss B., Georgopoulos M., Vass C., Schmetterer L. (2000). Assessment of optic disk blood flow in patients with open-angle glaucoma. Am. J. Ophthalmol..

[51] Schmidt K.G., von Rückmann A., Pillunat L.E. (1998). Topical carbonic anhydrase inhibition increases ocular pulse amplitude in high tension primary open angle glaucoma. Br. J. Ophthalmol..

[52] Kerr J., Nelson P., O’Brien C. (1998). A comparison of ocular blood flow in untreated primary open-angle glaucoma and ocular hypertension. Am. J. Ophthalmol..

[53] Fontana L., Poinoosawmy D., Bunce C.V., O’Brien C., Hitchings R.A. (1998). Pulsatile ocular blood flow investigation in asymmetric normal tension glaucoma and normal subjects. Br. J. Ophthalmol..

[54] Vulsteke C., Stalmans I., Fieuws S., Zeyen T. (2008). Correlation between ocular pulse amplitude measured by dynamic contour tonometer and visual field defects. Graefe’s Arch. Clin. Exp. Ophthalmol..

[55] Punjabi O.S., Ho H.-K.V., Kniestedt C., Bostrom A.G., Stamper R.L., Lin S.C. (2009). Intraocular Pressure and Ocular Pulse Amplitude Comparisons in Different Types of Glaucoma Using Dynamic Contour Tonometry. Curr. Eye Res..

[56] Kaufmann C., Bachmann L.M., Robert Y.C., Thiel M.A. (2006). Ocular Pulse Amplitude in Healthy Subjects as Measured by Dynamic Contour Tonometry. Arch. Ophthalmol..

[57] Schnichels S., Paquet-Durand F., Löscher M., Tsai T., Hurst J., Joachim S.C., Klettner A. (2021). Retina in a dish: Cell cultures, retinal explants and animal models for common diseases of the retina. Prog. Retin. Eye Res..

[58] Van Bergen N.J., Wood J.P.M., Chidlow G., Trounce I.A., Casson R.J., Ju W.-K., Weinreb R.N., Crowston J.G. (2009). Recharacterization of the RGC-5 Retinal Ganglion Cell Line. Investig. Ophthalmol. Vis. Sci..

[59] Krishnamoorthy R.R., Clark A.F., Daudt D., Vishwanatha J.K., Yorio T. (2013). A Forensic Path to RGC-5 Cell Line Identification: Lessons Learned. Investig. Ophthalmol. Vis. Sci..

[60] Ben-Ze’ev A., Robinson G.S., Bucher N.L., Farmer S.R. (1988). Cell-cell and cell-matrix interactions differentially regulate the expression of hepatic and cytoskeletal genes in primary cultures of rat hepatocytes. Proc. Natl. Acad. Sci. USA.

[61] Chen M.B., Srigunapalan S., Wheeler A.R., Simmons C.A. (2013). A 3D microfluidic platform incorporating methacrylated gelatin hydrogels to study physiological cardiovascular cell-cell interactions. Lab Chip.

[62] Park J., Koito H., Li J., Han A. (2009). Microfluidic compartmentalized co-culture platform for CNS axon myelination research. Biomed. Microdevices.

[63] Na K., Lee M., Shin H.-W., Chung S. (2017). In vitro nasal mucosa gland-like structure formation on a chip. Lab Chip.

[64] Biermann J., Boyle J., Pielen A., Lagrèze W.A. (2011). Histone deacetylase inhibitors sodium butyrate and valproic acid delay spontaneous cell death in purified rat retinal ganglion cells. Mol. Vis..

[65] Gao F., Li T., Hu J., Zhou X., Wu J., Wu Q. (2016). Comparative analysis of three purification protocols for retinal ganglion cells from rat. Mol. Vis..

[66] Nafian F., Kamali Doust Azad B., Yazdani S., Rasaee M.J., Daftarian N. (2020). A lab-on-a-chip model of glaucoma. Brain Behav..

[67] Tezel G., Li L.Y., Patil R.V., Wax M.B. (2001). TNF-alpha and TNF-alpha receptor-1 in the retina of normal and glaucomatous eyes. Investig. Ophthalmol. Vis. Sci..

[68] Yuan L., Neufeld A.H. (2000). Tumor necrosis factor-alpha: A potentially neurodestructive cytokine produced by glia in the human glaucomatous optic nerve head. Glia.

[69] Tsantoulas C., Farmer C., Machado P., Baba K., McMahon S.B., Raouf R. (2013). Probing Functional Properties of Nociceptive Axons Using a Microfluidic Culture System. PLoS ONE.

[70] Bennet D., Estlack Z., Reid T., Kim J. (2018). A microengineered human corneal epithelium-on-a-chip for eye drops mass transport evaluation. Lab Chip.

[71] Yu Z., Hao R., Du J., Wu X., Chen X., Zhang Y., Li W., Gu Z., Yang H. (2022). A human cornea-on-a-chip for the study of epithelial wound healing by extracellular vesicles. iScience.

[72] Bai J., Fu H., Bazinet L., Birsner A.E., D’Amato R.J. (2020). A Method for Developing Novel 3D Cornea-on-a-Chip Using Primary Murine Corneal Epithelial and Endothelial Cells. Front. Pharmacol..

[73] Deng Y., Li L., Xu J., Yao Y., Ding J., Wang L., Luo C., Yang W., Li L. (2024). A biomimetic human disease model of bacterial keratitis using a cornea-on-a-chip system. Biomater. Sci..

[74] Chung M., Lee S., Lee B.J., Son K., Jeon N.L., Kim J.H. (2017). Wet-AMD on a Chip: Modeling Outer Blood-Retinal Barrier In Vitro. Adv. Healthc. Mater..

[75] Arık Y.B., Buijsman W., Loessberg-Zahl J., Cuartas-Vélez C., Veenstra C., Logtenberg S., Grobbink A.M., Bergveld P., Gagliardi G., den Hollander A.I. (2021). Microfluidic organ-on-a-chip model of the outer blood–retinal barrier with clinically relevant read-outs for tissue permeability and vascular structure. Lab Chip.

[76] Kim J., Song Y., Jolly A.L., Hwang T., Kim S., Lee B., Jang J., Jo D.H., Baek K., Liu T.L. (2024). High-Throughput Microfluidic 3D Outer Blood-Retinal Barrier Model in a 96-Well Format: Analysis of Cellular Interactions and Barrier Function in Retinal Health and Disease. Adv. Mater. Technol..

[77] Maurissen T.L., Spielmann A.J., Schellenberg G., Bickle M., Vieira J.R., Lai S.Y., Pavlou G., Fauser S., Westenskow P.D., Kamm R.D. (2024). Modeling early pathophysiological phenotypes of diabetic retinopathy in a human inner blood-retinal barrier-on-a-chip. Nat. Commun..

[78] Leung C.M., de Haan P., Ronaldson-Bouchard K., Kim G.-A., Ko J., Rho H.S., Chen Z., Habibovic P., Jeon N.L., Takayama S. (2022). A guide to the organ-on-a-chip. Nat. Rev. Methods Primers.

[79] Park T.-E., Mustafaoglu N., Herland A., Hasselkus R., Mannix R., FitzGerald E.A., Prantil-Baun R., Watters A., Henry O., Benz M. (2019). Hypoxia-enhanced Blood-Brain Barrier Chip recapitulates human barrier function and shuttling of drugs and antibodies. Nat. Commun..

[80] Vatine G.D., Barrile R., Workman M.J., Sances S., Barriga B.K., Rahnama M., Barthakur S., Kasendra M., Lucchesi C., Kerns J. (2019). Human iPSC-Derived Blood-Brain Barrier Chips Enable Disease Modeling and Personalized Medicine Applications. Cell Stem Cell.

[81] Nair A.L., Groenendijk L., Overdevest R., Fowke T.M., Annida R., Mocellin O., de Vries H.E., Wevers N.R. (2023). Human BBB-on-a-chip reveals barrier disruption, endothelial inflammation, and T cell migration under neuroinflammatory conditions. Front. Mol. Neurosci..

[82] Kim J., Lee K.-T., Lee J.S., Shin J., Cui B., Yang K., Choi Y.S., Choi N., Lee S.H., Lee J.-H. (2021). Fungal brain infection modelled in a human-neurovascular-unit-on-a-chip with a functional blood–brain barrier. Nat. Biomed. Eng..

[83] Kim J., Koo B.-K., Knoblich J.A. (2020). Human organoids: Model systems for human biology and medicine. Nat. Rev. Mol. Cell Biol..

[84] Liu X., Zhou Z., Zhang Y., Zhong H., Cai X., Guan R. (2025). Recent progress on the organoids: Techniques, advantages and applications. Biomed. Pharmacother..

[85] Cho A.-N., Jin Y., An Y., Kim J., Choi Y.S., Lee J.S., Kim J., Choi W.-Y., Koo D.-J., Yu W. (2021). Microfluidic device with brain extracellular matrix promotes structural and functional maturation of human brain organoids. Nat. Commun..

[86] Salmon I., Grebenyuk S., Abdel Fattah A.R., Rustandi G., Pilkington T., Verfaillie C., Ranga A. (2022). Engineering neurovascular organoids with 3D printed microfluidic chips. Lab Chip.

[87] Quintard C., Tubbs E., Jonsson G., Jiao J., Wang J., Werschler N., Laporte C., Pitaval A., Bah T.-S., Pomeranz G. (2024). A microfluidic platform integrating functional vascularized organoids-on-chip. Nat. Commun..

[88] Cai H., Tian C., Chen L., Yang Y., Sun A.X., McCracken K., Tchieu J., Gu M., Mackie K., Guo F. (2025). Vascular network-inspired diffusible scaffolds for engineering functional midbrain organoids. Cell Stem Cell.

[89] Grebenyuk S., Abdel Fattah A.R., Kumar M., Toprakhisar B., Rustandi G., Vananroye A., Salmon I., Verfaillie C., Grillo M., Ranga A. (2023). Large-scale perfused tissues via synthetic 3D soft microfluidics. Nat. Commun..

[90] Osaki T., Duenki T., Chow S.Y.A., Ikegami Y., Beaubois R., Levi T., Nakagawa-Tamagawa N., Hirano Y., Ikeuchi Y. (2024). Complex activity and short-term plasticity of human cerebral organoids reciprocally connected with axons. Nat. Commun..

[91] Tsai Y.-C., Ozaki H., Morikawa A., Shiraiwa K., Pin A.P., Salem A.G., Phommahasay K.A., Sugita B.K., Vu C.H., Hammad S.M. (2025). Proof of concept for brain organoid-on-a-chip to create multiple domains in forebrain organoids. RSC Adv..

[92] Moshksayan K., Harihara A., Mondal S., Hegarty E., Atherly T., Sahoo D.K., Jergens A.E., Mochel J.P., Allenspach K., Zoldan J. (2023). OrganoidChip facilitates hydrogel-free immobilization for fast and blur-free imaging of organoids. Sci. Rep..

[93] Yamamoto K., Yamaoka N., Imaizumi Y., Nagashima T., Furutani T., Ito T., Okada Y., Honda H., Shimizu K. (2021). Development of a human neuromuscular tissue-on-a-chip model on a 24-well-plate-format compartmentalized microfluidic device. Lab Chip.

[94] Duc P., Vignes M., Hugon G., Sebban A., Carnac G., Malyshev E., Charlot B., Rage F. (2021). Human neuromuscular junction on micro-structured microfluidic devices implemented with a custom micro electrode array (MEA). Lab Chip.

[95] Gatti M., Dittlau K.S., Beretti F., Yedigaryan L., Zavatti M., Cortelli P., Palumbo C., Bertucci E., Van Den Bosch L., Sampaolesi M. (2023). Human Neuromuscular Junction on a Chip: Impact of Amniotic Fluid Stem Cell Extracellular Vesicles on Muscle Atrophy and NMJ Integrity. Int. J. Mol. Sci..

[96] Stoklund Dittlau K., Krasnow E.N., Fumagalli L., Vandoorne T., Baatsen P., Kerstens A., Giacomazzi G., Pavie B., Rossaert E., Beckers J. (2021). Human motor units in microfluidic devices are impaired by FUS mutations and improved by HDAC6 inhibition. Stem Cell Rep..

[97] Bonneau N., Potey A., Blond F., Guerin C., Baudouin C., Peyrin J.-M., Brignole-Baudouin F., Réaux-Le Goazigo A. (2024). Assessment of corneal nerve regeneration after axotomy in a compartmentalized microfluidic chip model with automated 3D high resolution live-imaging. Front. Cell. Neurosci..

[98] Bonneau N., Potey A., Vitoux M.-A., Magny R., Guerin C., Baudouin C., Peyrin J.-M., Brignole-Baudouin F., Réaux-Le Goazigo A. (2023). Corneal neuroepithelial compartmentalized microfluidic chip model for evaluation of toxicity-induced dry eye. Ocul. Surf..

[99] Li Q., Wong H.L., Ip Y.L., Chu W.Y., Li M.S., Saha C., Shih K.C., Chan Y.K. (2023). Current microfluidic platforms for reverse engineering of cornea. Mater. Today Bio.

[100] Abdalkader R., Kamei K.-i. (2020). Multi-corneal barrier-on-a-chip to recapitulate eye blinking shear stress forces. Lab Chip.

[101] Kado Abdalkader R., Chaleckis R., Fujita T., Kamei K.-i. (2024). Modeling dry eye with an air–liquid interface in corneal epithelium-on-a-chip. Sci. Rep..

[102] Seo J., Byun W.Y., Alisafaei F., Georgescu A., Yi Y.-S., Massaro-Giordano M., Shenoy V.B., Lee V., Bunya V.Y., Huh D. (2019). Multiscale reverse engineering of the human ocular surface. Nat. Med..

[103] Achberger K., Probst C., Haderspeck J., Bolz S., Rogal J., Chuchuy J., Nikolova M., Cora V., Antkowiak L., Haq W. (2019). Merging organoid and organ-on-a-chip technology to generate complex multi-layer tissue models in a human retina-on-a-chip platform. eLife.

[104] Gong J., Gong Y., Zou T., Zeng Y., Yang C., Mo L., Kang J., Fan X., Xu H., Yang J. (2023). A controllable perfusion microfluidic chip for facilitating the development of retinal ganglion cells in human retinal organoids. Lab Chip.

[105] Drabbe E., Pelaez D., Agarwal A. (2025). Retinal organoid chip: Engineering a physiomimetic oxygen gradient for optimizing long term culture of human retinal organoids. Lab Chip.

[106] Esteban-Linares A., Wareham L.K., Walmsley T.S., Holden J.M., Fitzgerald M.L., Pan Z., Xu Y.-Q., Li D. (2022). Dynamic Observation of Retinal Response to Pressure Elevation in a Microfluidic Chamber. Anal. Chem..

[107] Wheeler E.L., Stamer D.W., Au S., Overby D.R. (2024). Co-culture with trabecular meshwork cells promotes barrier function in an organ-on-chip model of Schlemm’s canal inner endothelial wall. Investig. Ophthalmol. Vis. Sci..

[108] Wheeler E.L., Stamer D.W., Wan Z., Kamm R., Au S., Overby D.R. (2023). Building an Organ-on-Chip Model of the Inner Wall Endothelium of Schlemm’s Canal. Investig. Ophthalmol. Vis. Sci..

[109] Lu R., Kolarzyk A.M., Stamer W.D., Lee E. (2025). Human ocular fluid outflow on-chip reveals trabecular meshwork-mediated Schlemm’s canal endothelial dysfunction in steroid-induced glaucoma. Nat. Cardiovasc. Res..

[110] Coombs J., van der List D., Wang G.Y., Chalupa L.M. (2006). Morphological properties of mouse retinal ganglion cells. Neuroscience.

[111] Goldberg J.L., Espinosa J.S., Xu Y., Davidson N., Kovacs G.T.A., Barres B.A. (2002). Retinal Ganglion Cells Do Not Extend Axons by Default: Promotion by Neurotrophic Signaling and Electrical Activity. Neuron.

[112] Mak H.K., Yung J.S.Y., Weinreb R.N., Ng S.H., Cao X., Ho T.Y.C., Ng T.K., Chu W.K., Yung W.H., Choy K.W. (2020). MicroRNA-19a-PTEN Axis Is Involved in the Developmental Decline of Axon Regenerative Capacity in Retinal Ganglion Cells. Mol. Ther. Nucleic Acids.

[113] Catenaccio A., Llavero Hurtado M., Diaz P., Lamont D.J., Wishart T.M., Court F.A. (2017). Molecular analysis of axonal-intrinsic and glial-associated co-regulation of axon degeneration. Cell Death Dis..

[114] Taylor A.M., Blurton-Jones M., Rhee S.W., Cribbs D.H., Cotman C.W., Jeon N.L. (2005). A microfluidic culture platform for CNS axonal injury, regeneration and transport. Nat. Methods.

[115] Pagella P., Neto E., Jiménez-Rojo L., Lamghari M., Mitsiadis T.A. (2014). Microfluidics co-culture systems for studying tooth innervation. Front. Physiol..

[116] Nijssen J., Aguila J., Hoogstraaten R., Kee N., Hedlund E. (2018). Axon-Seq Decodes the Motor Axon Transcriptome and Its Modulation in Response to ALS. Stem Cell Rep..

[117] Bhattacharyya R., Black S.E., Lotlikar M.S., Fenn R.H., Jorfi M., Kovacs D.M., Tanzi R.E. (2021). Axonal generation of amyloid-β from palmitoylated APP in mitochondria-associated endoplasmic reticulum membranes. Cell Rep..

[118] Fujita Y., Nakanishi T., Ueno M., Itohara S., Yamashita T. (2020). Netrin-G1 Regulates Microglial Accumulation along Axons and Supports the Survival of Layer V Neurons in the Postnatal Mouse Brain. Cell Rep..

[119] Peng Z., Chen Y., Wu T. (2020). Ultrafast Microdroplet Generation and High-Density Microparticle Arraying Based on Biomimetic Nepenthes Peristome Surfaces. ACS Appl. Mater. Interfaces.

[120] Lee S., Van Bergen N.J., Kong G.Y., Chrysostomou V., Waugh H.S., O’Neill E.C., Crowston J.G., Trounce I.A. (2011). Mitochondrial dysfunction in glaucoma and emerging bioenergetic therapies. Exp. Eye Res..

[121] Kong G.Y., Van Bergen N.J., Trounce I.A., Crowston J.G. (2009). Mitochondrial dysfunction and glaucoma. J. Glaucoma.

[122] Wilmer M.J., Ng C.P., Lanz H.L., Vulto P., Suter-Dick L., Masereeuw R. (2016). Kidney-on-a-Chip Technology for Drug-Induced Nephrotoxicity Screening. Trends Biotechnol..

[123] Bogdanowicz D.R., Lu H.H. (2014). Multifunction co-culture model for evaluating cell-cell interactions. Methods Mol. Biol..

[124] Goers L., Freemont P., Polizzi K.M. (2014). Co-culture systems and technologies: Taking synthetic biology to the next level. J. R. Soc. Interface.

[125] Mi S., Du Z., Xu Y., Wu Z., Qian X., Zhang M., Sun W. (2016). Microfluidic co-culture system for cancer migratory analysis and anti-metastatic drugs screening. Sci. Rep..

[126] Aires I.D., Boia R., Rodrigues-Neves A.C., Madeira M.H., Marques C., Ambrósio A.F., Santiago A.R. (2019). Blockade of microglial adenosine A(2A) receptor suppresses elevated pressure-induced inflammation, oxidative stress, and cell death in retinal cells. Glia.

[127] Gensheimer T., Veerman D., van Oosten E.M., Segerink L., Garanto A., van der Meer A.D. (2025). Retina-on-chip: Engineering functional in vitro models of the human retina using organ-on-chip technology. Lab Chip.

[128] Ho K.K.Y., Wang Y.L., Wu J., Liu A.P. (2018). Advanced Microfluidic Device Designed for Cyclic Compression of Single Adherent Cells. Front. Bioeng. Biotechnol..

[129] Rosser J., Olmos Calvo I., Peter E., Jenner F., Purtscher M., Shlager M. (2015). Recent Advances of Biologically Inspired 3D Microfluidic Hydrogel Cell Culture Systems. J. Cell Biol. Cell Metab..

[130] Chang M.Y., Shin A., Park J., Nagiel A., Lalane R.A., Schwartz S.D., Demer J.L. (2017). Deformation of Optic Nerve Head and Peripapillary Tissues by Horizontal Duction. Am. J. Ophthalmol..

[131] Fazio M.A., Clark M.E., Bruno L., Girkin C.A. (2018). In vivo optic nerve head mechanical response to intraocular and cerebrospinal fluid pressure: Imaging protocol and quantification method. Sci. Rep..

[132] Downs J.C., Girkin C.A. (2017). Lamina cribrosa in glaucoma. Curr. Opin. Ophthalmol..

[133] Tian H., Li L., Song F. (2017). Study on the deformations of the lamina cribrosa during glaucoma. Acta Biomater..

[134] Jin Y., Wang X., Zhang L., Jonas J.B., Aung T., Schmetterer L., Girard M.J.A. (2018). Modeling the Origin of the Ocular Pulse and Its Impact on the Optic Nerve Head. Investig. Ophthalmol. Vis. Sci..

[135] Braunsmann C., Hammer C.M., Rheinlaender J., Kruse F.E., Schäffer T.E., Schlötzer-Schrehardt U. (2012). Evaluation of Lamina Cribrosa and Peripapillary Sclera Stiffness in Pseudoexfoliation and Normal Eyes by Atomic Force Microscopy. Investig. Ophthalmol. Vis. Sci..

[136] Liu J.H.K., Zhang X., Kripke D.F., Weinreb R.N. (2003). Twenty-four-Hour Intraocular Pressure Pattern Associated with Early Glaucomatous Changes. Investig. Ophthalmol. Vis. Sci..

[137] De Smedt S. (2015). Noninvasive intraocular pressure monitoring: Current insights. Clin. Ophthalmol..

[138] Shajiei T.D., Iadanza S., Bachmann L.M., Kniestedt C. (2024). Inventory of Ocular Pulse Amplitude Values in Healthy Subjects and Patients With Ophthalmologic Illnesses: Systematic Review and Meta-analysis. Am. J. Ophthalmol..

[139] Cheng L., Ding Y., Duan X., Wu Z. (2017). Ocular pulse amplitude in different types of glaucoma using dynamic contour tonometry: Diagnosis and follow-up of glaucoma. Exp. Ther. Med..

[140] Edwards M.E., Wang S.S., Good T.A. (2001). Role of viscoelastic properties of differentiated SH-SY5Y human neuroblastoma cells in cyclic shear stress injury. Biotechnol. Prog..

[141] Voorhees A.P., Jan N.J., Sigal I.A. (2017). Effects of collagen microstructure and material properties on the deformation of the neural tissues of the lamina cribrosa. Acta Biomater..

[142] Li L., Song F. (2020). Biomechanical research into lamina cribrosa in glaucoma. Natl. Sci. Rev..

[143] Yoshikawa M., Akagi T., Hangai M., Ohashi-Ikeda H., Takayama K., Morooka S., Kimura Y., Nakano N., Yoshimura N. (2014). Alterations in the Neural and Connective Tissue Components of Glaucomatous Cupping After Glaucoma Surgery Using Swept-Source Optical Coherence Tomography. Investig. Ophthalmol. Vis. Sci..

[144] Ma Y., Kwok S., Sun J., Pan X., Pavlatos E., Clayson K., Hazen N., Liu J. (2020). IOP-induced regional displacements in the optic nerve head and correlation with peripapillary sclera thickness. Exp. Eye Res..

[145] Hallfors N., Khan A., Dickey M.D., Taylor A.M. (2013). Integration of pre-aligned liquid metal electrodes for neural stimulation within a user-friendly microfluidic platform. Lab Chip.

[146] Ronchi S., Fiscella M., Marchetti C., Viswam V., Müller J., Frey U., Hierlemann A. (2019). Single-Cell Electrical Stimulation Using CMOS-Based High-Density Microelectrode Arrays. Front. Neurosci..

[147] Gupta P., Shinde A., Illath K., Kar S., Nagai M., Tseng F.-G., Santra T.S. (2022). Microfluidic platforms for single neuron analysis. Mater. Today Bio.

[148] Duvan F.T., Cunquero M., Masvidal-Codina E., Walston S.T., Marsal M., de la Cruz J.M., Viana D., Nguyen D., Degardin J., Illa X. (2024). Graphene-based microelectrodes with bidirectional functionality for next-generation retinal electronic interfaces. Nanoscale Horiz..

[149] Kim D.E., Kim S., Kim M., Min B.-K., Im M. (2025). Retinal degeneration increases inter-trial variabilities of light-evoked spiking activities in ganglion cells. Exp. Eye Res..

[150] Fiscella M., Farrow K., Jones I.L., Jäckel D., Müller J., Frey U., Bakkum D.J., Hantz P., Roska B., Hierlemann A. (2012). Recording from defined populations of retinal ganglion cells using a high-density CMOS-integrated microelectrode array with real-time switchable electrode selection. J. Neurosci. Methods.

[151] Sibille J., Gehr C., Benichov J.I., Balasubramanian H., Teh K.L., Lupashina T., Vallentin D., Kremkow J. (2022). High-density electrode recordings reveal strong and specific connections between retinal ganglion cells and midbrain neurons. Nat. Commun..

[152] Zhang K., Liu Y., Song Y., Xu S., Yang Y., Jiang L., Sun S., Luo J., Wu Y., Cai X. (2023). Exploring retinal ganglion cells encoding to multi-modal stimulation using 3D microelectrodes arrays. Front. Bioeng. Biotechnol..

[153] Hong G., Fu T.-M., Qiao M., Viveros R.D., Yang X., Zhou T., Lee J.M., Park H.-G., Sanes J.R., Lieber C.M. (2018). A method for single-neuron chronic recording from the retina in awake mice. Science.

[154] Tran N.M., Shekhar K., Whitney I.E., Jacobi A., Benhar I., Hong G., Yan W., Adiconis X., Arnold M.E., Lee J.M. (2019). Single-Cell Profiles of Retinal Ganglion Cells Differing in Resilience to Injury Reveal Neuroprotective Genes. Neuron.

[155] Eickenscheidt M., Zeck G. (2014). Action potentials in retinal ganglion cells are initiated at the site of maximal curvature of the extracellular potential. J. Neural Eng..

[156] Szu-Yu Ho T., Rasband M.N. (2011). Maintenance of neuronal polarity. Dev. Neurobiol..

[157] Kole M.H., Stuart G.J. (2012). Signal Processing in the Axon Initial Segment. Neuron.

[158] Ko K.W., Rasband M.N., Meseguer V., Kramer R.H., Golding N.L. (2016). Serotonin modulates spike probability in the axon initial segment through HCN channels. Nat. Neurosci..

[159] Obien M.E.J., Deligkaris K., Bullmann T., Bakkum D.J., Frey U. (2015). Revealing neuronal function through microelectrode array recordings. Front. Neurosci..

[160] Kim R., Joo S., Jung H., Hong N., Nam Y. (2014). Recent trends in microelectrode array technology for in vitro neural interface platform. Biomed. Eng. Lett..

[161] Kobayashi T., Shimba K., Narumi T., Asahina T., Kotani K., Jimbo Y. (2024). Revealing single-neuron and network-activity interaction by combining high-density microelectrode array and optogenetics. Nat. Commun..

[162] Claverol-Tinture E., Cabestany J., Rosell X. (2007). Multisite Recording of Extracellular Potentials Produced by Microchannel-Confined Neurons In-Vitro. IEEE Trans. Biomed. Eng..

[163] Habibey R., Latifi S., Mousavi H., Pesce M., Arab-Tehrany E., Blau A. (2017). A multielectrode array microchannel platform reveals both transient and slow changes in axonal conduction velocity. Sci. Rep..

[164] Cogan S.F., Troyk P.R., Ehrlich J., Plante T.D. (2005). In Vitro Comparison of the Charge-Injection Limits of Activated Iridium Oxide (AIROF) and Platinum-Iridium Microelectrodes. IEEE Trans. Biomed. Eng..

